# Interactions between islet-resident macrophages and β cells in diabetes

**DOI:** 10.3389/fimmu.2025.1630507

**Published:** 2025-07-28

**Authors:** Danhuai Zhang, Lingzhe Meng, Minghui Xi, Shuai Li, Wantong Chen, LuYi Li, Lingling Dong, Na Wu

**Affiliations:** ^1^ Department of Pediatrics, Shengjing Hospital of China Medical University, Shenyang, China; ^2^ Department of Public Health, Shengjing Hospital of China Medical University, Shenyang, China; ^3^ Department of Thoracic Surgery, Shengjing Hospital of China Medical University, Shenyang, China; ^4^ Department of Emergency, Shengjing Hospital of China Medical University, Shenyang, China; ^5^ Department of Cardiology, Shengjing Hospital of China Medical University, Shenyang, China

**Keywords:** islet-resident macrophages, β cells, diabetes, cell-cell interaction, immunometabolism, therapeutic targets

## Abstract

In diabetes, islet-resident macrophages (IRMs) and β cells engage in multifaceted interactions through diverse signaling pathways and cell–cell contact within the islet microenvironment, jointly shaping both homeostasis and disease progression. This review first outlines the origin, renewal dynamics, and phenotypic heterogeneity of IRMs, highlighting their essential roles in maintaining metabolic and immunological homeostasis under physiological conditions. We then emphasize the dual role of IRMs in type 1 and type 2 diabetes (T1DM and T2DM): in T1DM, they drive autoimmunity via antigen presentation and pro-inflammatory cytokine secretion; in T2DM, metabolic stress induces M1 polarization, exacerbating β cell dysfunction and dedifferentiation. We further explore molecular mechanisms modulating IRM–β cell crosstalk, including neuro-immune-endocrine networks (e.g., α1-adrenergic signaling), Interleukin-1 Beta (IL-1β) feedback loops, and the C-X-C Motif Chemokine Ligand 16 (CXCL16)/Oxidized Low-Density Lipoprotein (OxLDL) axis. The paracrine actions of growth factors such as PDGF, VEGF-A, and IGF-1 in β cell proliferation and regeneration are also reviewed. Additionally, novel therapeutic targets, such as G Protein-Coupled Receptor 132 (GPR132) and exosomal miRNAs, offer promising strategies to precisely regulate macrophage polarization and protect β cells. Finally, we discuss the application of advanced technologies—such as single-cell sequencing and intravital imaging—in deciphering dynamic IRM–β cell interactions and highlight the prospects of modulating islet macrophage phenotypes to restore metabolic and immune balance in future research and clinical translation.

## 1 Introduction

Diabetes is a metabolic disease that poses a serious threat to global health. It is characterized by insufficient insulin secretion or islet dysfunction, resulting in the inability of the body to regulate blood glucose levels effectively. As one of the most prevalent non-communicable diseases worldwide, the International Diabetes Federation’s IDF Diabetes Atlas (10th edition, 2021) estimated that approximately 537 million adults (aged 20–79) were living with diabetes in 2021. This number is projected to rise to 643 million by 2030 and reach 783 million by 2045 (1).

Recent studies have highlighted that the dynamic interactions between islet-resident macrophages (IRMs) and β cells play a central role in the pathophysiology of diabetes (2, 3). The islet microenvironment represents a complex immunoendocrine network, the homeostasis of which relies on the precise regulation of multiple cell types. As key components of the innate immune system, IRMs participate in regulating β-cell function and fate through various mechanisms. Under physiological conditions, two distinct macrophage subsets are found in islets: resident macrophages (CD68^+^F4/80^+^) and recruited macrophages (CD68^+^F4/80^-^). In diabetic mouse models, the number of islet macrophages significantly increases—resident macrophages expand by fourfold and recruited macrophages by eightfold. These macrophages predominantly adopt a pro-inflammatory M1-like phenotype, closely associated with disease progression (4). Notably, IRMs exhibit a unique spatial distribution, forming close anatomical proximity with β cells, providing a structural basis for their functional interactions (2, 3, 5).

In T1DM, IRMs amplify T-cell infiltration through α1-adrenergic receptor-mediated neuroimmune signaling (5, 6), whereas in T2DM, distinct IRM subsets diverge into β-cell-damaging or proliferative-promoting macrophages, resulting in a functional paradox (7, 8). Recent findings suggest that the bidirectional feedback loop of IL-1β (rapid pro-inflammatory *vs*. delayed anti-inflammatory) and the immune-metabolic imbalance mediated by the CXCL16/OxLDL axis are critical determinants of β-cell survival (9, 10).

However, most mechanistic insights to date have been derived from murine models, which, while invaluable for exploring disease mechanisms, inherently represent simplified systems that cannot fully capture the genetic, immunological, and phenotypic heterogeneity of human diabetes (11, 12). Moreover, the predominant use of single mouse strains in gene knockout or transgenic studies limits the generalizability of their conclusions. Pathological studies have shown significant heterogeneity in pancreatic alterations across patients with diabetes onset at different ages (13–19), suggesting that observations in murine models may not be universally applicable across all clinical subtypes. Thus, although this review synthesizes extensive research progress primarily based on animal models, these findings must be extrapolated to human diabetes with caution.

This review aims to systematically elucidate the molecular mechanisms and pathological significance of IRM–β-cell interactions in diabetes. Key scientific questions addressed include: (1) the heterogeneity and plasticity of IRMs within the islet microenvironment; (2) the dual roles of IRM–β-cell interactions in the initiation and progression of diabetes; (3) regulatory mechanisms based on the neuro-immune-endocrine network; and (4) potential therapeutic targets. By integrating recent advances, this work provides new perspectives for understanding diabetes pathogenesis and lays the theoretical foundation for developing islet microenvironment-targeted intervention strategies (3, 20, 21).

## 2 IRMs involvement in β cell development and proliferation

IRMs are closely associated with β-cell development (22). Derived from definitive hematopoiesis, adult IRMs originate from hematopoietic stem cells (HSCs). Experimental evidence using Flt3-Cre mouse models confirms their dependence on the FLT3-expressing HSC lineage and not on primitive hematopoiesis, such as that arising from the yolk sac (23). In contrast, embryonic macrophages originate from yolk sac hematopoiesis, reside in the fetal pancreas, and exhibit tissue-residency properties that support endocrine cell differentiation and vascularization (24). This distinction is essential for coordinating developmental roles at different life stages. Studies have shown that the number of IRMs directly affects β-cell numbers. In CSF-1-deficient op/op mice, macrophage development is impaired, resulting in abnormal islet formation and reduced β-cell proliferation, with significantly decreased β-cell numbers, while α-cell counts remain unaffected. However, islet morphogenesis is nonetheless disrupted (22, 25–27).

The regulatory role of IRMs in β-cell regeneration exhibits spatial and temporal specificity. Under physiological conditions, adult β-cell proliferation is restricted by a low renewal rate, and this age-dependent decline is closely linked to the decreased activity of the platelet-derived growth factor (PDGF) signaling pathway. The PDGFRα/Erk/Ezh2 axis becomes inactivated with age, leading to upregulation of the cell cycle inhibitor p16^INK4a. Selective activation of PDGFRα restores β-cell proliferative capacity—resulting in a ninefold increase in proliferation while maintaining normal function. This pathway is also conserved in human islets (28). Under specific physiological or pathological conditions such as pregnancy and obesity, β cells can re-enter the cell cycle. In rodent models of obesity, β-cell mass exhibits adaptive expansion during the prediabetic stage (29–31), regulated by various factors including glucose metabolites (32, 33), insulin signaling (34), and hepatocyte growth factor (35, 36). Recent studies demonstrate that high-fat diet (HFD) induces significant upregulation of PDGFα in macrophages. Given that macrophages are a major source of PDGF (37–39), Ying et al. validated the involvement of this pathway, confirming that macrophage-derived PDGF activates PDGFR on β-cell surfaces and promotes proliferation in a dose-dependent manner (7). During the compensatory phase of diabetes (i.e., hyperinsulinemia), IRMs secrete VEGF-A to promote angiogenesis, supporting islet hypertrophy and increased vascular density, thereby maintaining β-cell insulin secretion. Experiments using chemical depletion (clodronate), genetic models (CD169-DTR mice), or antibody blockade (anti-CSF1R) showed that macrophage ablation results in reduced islet vascular density and volume, impaired insulin secretion, glucose intolerance, and the collapse of compensatory hyperinsulinemia. Mechanistic studies reveal that macrophages do not regulate β-cell genes such as insulin or PDX1 directly but instead act through a paracrine mechanism by releasing VEGF-A and inflammatory factors (e.g., IL-6) to promote angiogenesis and optimize the local microenvironment, thereby indirectly enhancing β-cell function (40). This mode of microenvironment-dependent regulation suggests that macrophages serve as pivotal nodes in the “vasculature–β-cell” interaction network during metabolic compensation.

Of note, external regulatory factors such as the gut microbiota may activate IRMs via pattern recognition receptors. For example, cell wall components from fungi such as C. dubliniensis can induce IRM infiltration and trigger β-cell regeneration in diabetic models, a process strictly dependent on macrophages (41). Similarly, Riley et al. explored the role of connective tissue growth factor (CTGF/CCN2) in inducing β-cell regeneration in a 50% β-cell loss mouse model (42). CTGF, secreted by vascular endothelial cells, is associated with extracellular matrix dynamics and upregulates β-cell cycle genes. Interestingly, CTGF also increased islet macrophage numbers, and its regenerative effects were completely abolished by clodronate sodium injection, indicating that macrophage presence is essential for CTGF-induced β-cell regeneration (42, 43).

Following β-cell death, IRMs remove apoptotic cells via efferocytosis and initiate repair programs. In this phase, pro-inflammatory cytokine expression (e.g., TNF, IL-1β) is downregulated, while IGF-1 and TGFBI are upregulated. Concurrently, metabolic activity, lysosomal function, and lipid metabolism pathways are significantly enhanced (42, 44). These dynamic changes are especially prominent during tissue repair. In the compensatory phase, IRMs exhibit an adaptive phenotype—characterized by elevated PDGF, VEGF, and IGF-1 expression and enhanced glycolysis through metabolic reprogramming (40, 42, 44). However, chronic glucolipotoxicity drives IRMs toward a pro-inflammatory M1 phenotype that secretes TNF-α and IL-1β, ultimately leading to β-cell dedifferentiation and collapse of compensatory mechanisms (45–49). This state of decompensation will be discussed further in later sections.Despite substantial evidence supporting IRMs’ role in β-cell regeneration, the spatiotemporal regulation of their signaling networks remains unclear. Key unresolved issues include the functional division among heterogeneous IRM subsets (e.g., CD11c^+^ *vs*. CD68^+^), and how therapeutic phenotypic reprogramming may be achieved to prevent chronic inflammation. These will be critical directions for future translational research.

## 3 Other biological characteristics of IRMs

### 3.1 Phenotypic features and distribution

IRMs represent the only resident myeloid cell population within the islets (50). Phenotypically, they highly express major histocompatibility complex class II (MHC-II), CD11c, F4/80, CD11b, CD64, lysozyme, CX3C chemokine receptor 1 (CX3CR1), and CXCL16, while lacking expression of CD206 (mannose receptor) and CD301 (macrophage galactose-type C-type lectin) (10, 20, 47). Notably, IRMs constitute the predominant leukocyte population in both lean and obese mice, whereas their proportion is significantly lower in human islets (47, 51, 52). In obese mice, two distinct IRM subsets have been identified: intrainsular macrophages (high CD11c and MHC-II expression, directly contacting β cells) and perinsular macrophages (low CD11c, located in the islet capsule, more involved in local immune regulation) (7). Peri-islet macrophages may serve as a barrier to block infiltration by other immune cells or, under obese conditions, promote β-cell proliferation (53). RNA sequencing of IRMs from lean and obese mice revealed no clear shift along the M1/M2 spectrum, though their functional properties differ: intrainsular IRMs inhibit insulin secretion by uptaking secretory granules from β cells, while perinsular IRMs promote β-cell proliferation via PDGF/PDGFR signaling (7).

### 3.2 Renewal kinetics

Unlike macrophages derived from circulating monocytes, IRMs exhibit unique renewal kinetics. Under homeostasis, they show extremely low turnover with peripheral blood, maintaining their population via local self-renewal (7, 47, 54, 55). Transcriptionally, they display a resting phenotype—low expression of pro-inflammatory and M2 markers. During inflammation, however, bone marrow-derived monocytes may be recruited to replenish IRMs through local maturation (56, 57).

With aging, IRMs are gradually replaced by bone marrow–derived monocytes, independent of the CCL2/CCR2 axis. In contrast, exocrine macrophages marked by Tim-4^-^MHCII^+^ are replaced in a CCR2-dependent manner. Interestingly, obesity and T2DM do not significantly alter the turnover dynamics of pancreatic macrophage subsets (58). According to Zirpel and Roep (2021), such replacement properties suggest that IRMs may serve as key mediators linking stressed β cells with the activation of the adaptive immune system (20).

Following lethal irradiation, IRMs can be fully replaced by donor bone marrow–derived cells and retain M1-like activation (e.g., high MHC-II, IL-1β, TNF-α expression) (47). However, their precise distribution remains undefined. Using adoptive transfer, Ying et al. showed that fluorescently labeled Ly6C^+^ monocytes localized to the pancreas–exocrine interface but did not infiltrate the islets in obese mice. These transferred cells retained their monocyte phenotype and did not differentiate into macrophages within the pancreas (7).

### 3.3 Polarization states and phagocytic capacity

IRMs exhibit tissue-specific polarization states. Parv et al. (2021) demonstrated that IRMs in mice predominantly exhibit a pro-inflammatory M1-like phenotype, while exocrine tissue macrophages tend toward an alternatively activated M2-like state. This difference may be associated with local environmental influence rather than intrinsic phagocytic gene expression, as both populations show similar expression of phagocytosis-related genes (59).

Functionally, endocrine IRMs have superior phagocytic and endocytic capacities both *in vitro* and *in vivo* compared to exocrine macrophages (59). Enhanced phagocytosis may drive macrophages toward an inflammatory phenotype (60), suggesting that phagocytic burden could act as an environmental signal promoting M1 polarization. Of note, Srivastava et al. (2024) revealed that CXCL16 deficiency alters OxLDL clearance and significantly affects the survival and function of transitional CD8^+^ T cells within the islet, emphasizing the role of polarization in autoimmune diabetes (10).

### 3.4 Functional adaptability

IRMs demonstrate high adaptability to metabolic cues within the islet microenvironment. In obesity, elevated glucose and fatty acids can stimulate the release of proliferative factors, promoting local expansion of IRMs (61–63). Weitz et al. (2018) discovered that IRMs express functional purinergic receptors, enabling them to detect ATP released by β cells upon glucose stimulation. ATP binding increases intracellular Ca²^+^ and enhances pseudopodia dynamics, allowing IRMs to monitor endocrine activity (64). While this serves as a metabolic surveillance function under normal conditions, stress-induced ATP in obesity, aging, or T2DM acts as a damage-associated molecular pattern (DAMP), triggering NLRP3 inflammasome activation and IL-1β release, along with upregulation of MHC-II and costimulatory molecules (65, 66). Thus, β-cell-derived signals not only mediate metabolic coordination but may also flag “abnormal” β cells to the immune system. As Kapetanovic et al. noted, chronic accumulation of DAMPs like ATP can become “immunological ticking time bombs,” driving innate immune overactivation and chronic inflammation in aging and disease (65).

Additionally, IRMs establish specialized spatial relationships with β-cells, enabling them to precisely sense β-cell secretory activity and viability (2). The study by Wei Ying et al. provides functional evidence that under obesity, IRMs directly impair insulin vesicle release through contact-dependent phagocytosis. Ultrastructural analyses reveal that macrophages capture intact insulin secretory granules adjacent to the β-cell plasma membrane. Combined with functional experiments, this suggests that IRMs likely phagocytose docked yet unreleased vesicles via cell-contact mechanisms (7). These findings indicate that IRMs develop distinct functional specialization by adapting to the islet microenvironment, with their functional focus potentially shifting from immune defense toward maintaining local metabolic homeostasis and inflammatory balance.

## 4 Functional and immunomodulatory properties of β cells

As the principal insulin-secreting cells in the pancreatic islets, β cells are closely integrated with the immune microenvironment, and their functional states are dynamically regulated by immune cues. Recent evidence suggests that β cells not only regulate glucose metabolism but also actively participate in crosstalk with immune cells, particularly macrophages, via multiple bioactive factors.

Studies in animal models indicate that islet β cells possess strong adaptive plasticity under metabolic and immune stress. In high-fat diet (HFD)-induced T2DM models, the increase in β-cell mass is primarily achieved through self-replication rather than transdifferentiation from other endocrine cells. In contrast, in STZ-induced T1DM models, β cells exhibit prominent dedifferentiation and transdifferentiation into α/δ cells, with partial reverse conversion back to β cells during recovery. Single-cell RNA sequencing (scRNA-seq) has further elucidated the molecular mechanisms underlying these transitions, highlighting roles for mitochondrial dynamics, chromatin remodeling, and epigenetic regulation in β-cell fate decisions (67).

Under physiological conditions, β cells maintain glucose homeostasis through tightly regulated insulin secretion. Zinselmeyer et al. (2018), using two-photon microscopy, observed that IRMs form intimate spatial contacts with β cells, extending filopodia into blood vessels and capturing secreted insulin granules. These macrophages can then present insulin-derived peptides to CD4^+^ T cells (66). Crucially, this antigen presentation does not activate naïve CD4^+^ T cells but instead expands previously activated effector Th1 cells, suggesting a role in local autoimmune amplification rather than initiation—particularly relevant in the context of T1DM (66).

In obesity and T2DM, the β-cell secretory profile changes markedly. Mukhuty et al. showed that both the MIN6 β-cell line and isolated islets secrete fetuin-A, which drives macrophage M1 polarization and enhances the expression of Emr1, CD68, and CD11c, along with chemotactic recruitment to the islets. This shift impairs insulin secretion and promotes local inflammation (68). Moreover, β cells may regulate macrophage polarization through microRNAs, suggesting a novel avenue by which M1 macrophage-induced β-cell dysfunction may be mediated (69). In addition to insulin, β cells release several immunoregulatory mediators. Upon high-glucose stimulation, ATP stored within insulin granules is co-secreted with insulin (70, 71). Studies suggest that the Vesicular Nucleotide Transporter (VNUT) located on the membrane of β-cell secretory granules may be involved in both intra-granular ATP accumulation and its release (71–73). Furthermore, P2X7 receptors and pannexin-1 channels primarily regulate the release of pro-inflammatory cytokines induced by extracellular ATP during islet inflammation, whereas they do not directly mediate the ATP release process itself (72).

In summary, β cells contribute to shaping the islet immune microenvironment through multiple signaling molecules—including ATP, peptide antigens, microRNAs, and fetuin-A. These mediators influence macrophage polarization and antigen presentation, potentially driving β-cell dysfunction in obesity and autoimmunity. The following section will explore how these signaling pathways integrate with the progression of inflammation and activation of adaptive immunity.

## 5 Mechanisms of interaction between IRMs and β cells

The interactions between IRMs and β cells operate through two principal modes: direct physical contact and indirect modulation via soluble factors. Under normal conditions, IRMs maintain islet homeostasis by secreting anti-inflammatory cytokines such as IL-10 and TGF-β, thereby suppressing excessive immune activation (49). They also support β-cell proliferation and survival through key signaling cascades including Notch, Wnt/β-catenin, PI3K/Akt, and Erk1/2 pathways (74–77). In 2019, Chittezhath et al. reported that IRMs are crucial for islet vascular remodeling and play a central role in compensatory hyperinsulinemia during the early stages of insulin resistance. Dysregulation of IRM function accelerates diabetes progression, although the exact mechanisms remain incompletely understood (40). Under pathological conditions, including both type 1 and type 2 diabetes, homeostatic balance in the islet microenvironment is disrupted. IRMs become polarized toward a pro-inflammatory phenotype, resulting in elevated secretion of IL-1β and TNF-α (46–49). This shift contributes to a vicious cycle wherein the loss of anti-inflammatory cytokines and the sustained release of inflammatory mediators exacerbate β-cell damage. In turn, damaged β cells release danger signals that further activate IRMs, perpetuating local inflammation.

Recent organoid-based studies using human embryonic stem cell–derived islet–macrophage interface (VMI) models reveal that in the context of viral infections—such as SARS-CoV-2 or Coxsackievirus B4—IRMs can induce β-cell pyroptosis via inflammatory cytokines and the TNFSF12–TNFRSF12A axis, leading to accelerated islet dysfunction (78). These macrophages operate via dual mechanisms: (1) phagocytosis of apoptotic β cells leads to the internalization of insulin crystals, causing lysosomal swelling, membrane disruption, and subsequent reactive oxygen species (ROS) accumulation that activates the NLRP3 inflammasome and triggers massive IL-1β release (79–81); and (2) metabolic stress induces lipid accumulation in macrophages, forming foam cell–like phenotypes that continuously secrete inflammatory mediators. This β-cell death–macrophage activation feedback loop amplifies inflammation through JNK/NF-κB and MAPK/NF-κB pathways, ultimately resulting in β-cell failure (82, 83). Importantly, β cells are not merely passive targets; insulin-derived peptides (e.g., insB:9–23) can engage the atypical receptor Olfactory Receptor 109 (Olfr109) on β-cell surfaces. This Gi-coupled pathway suppresses insulin secretion and modulates IRM function via CCL2 production, forming a local inflammatory feedback loop anchored in direct cell–cell contact (84). However, Cheng et al. noted that insB:9–23 was detected from crude granule-related membrane fractions obtained by differential centrifugation, which may include secondary lysosomes, crinophagic bodies, or autophagosomes, rather than purified secretory granules. Therefore, the presence of proteases required for insB:9–23 production within functional insulin granules remains uncertain. While under pathological conditions such as inflammation these peptides may be released through the fusion of dysfunctional organelles with the plasma membrane, this hypothesis has yet to be confirmed experimentally (84).

Beyond direct macrophage–β-cell communication, β cells also respond to paracrine signals from other islet endocrine cells, particularly α and δ cells (85, 86). For example, under metabolic or inflammatory stress, α cells may release CXCL10 and other immunomodulatory cytokines that influence IRM activation and promote recruitment of CXCR3^+^ T cells and macrophages, exacerbating inflammation (87). δ-cell–derived somatostatin may also modulate IRM behavior and contribute to immunological balance (85). Interestingly, recent evidence suggests α cells may play critical roles in early T1DM inflammation. β cells located adjacent to α cells (“α-linked β cells”) are more likely to interact with T cells and macrophages, possibly due to higher or earlier expression of IL-1β and IL-6 by α cells, forming inflammatory hotspots within the islets (87). Moreover, β cells may undergo transdifferentiation into α/δ cells during STZ-induced T1DM, with reverse conversion aiding functional restoration, as previously described (67).

These observations suggest that IRM-mediated effects are not solely due to direct interaction with β cells but may also involve crosstalk with α and δ cells and indirect paracrine effects. This complex signaling network warrants deeper investigation to fully understand how IRMs orchestrate the broader immune–endocrine dynamics of the islet microenvironment.

### 5.1 Mechanisms of IRM–β cell interactions in T1DM development

During the development of type 1 diabetes mellitus (T1DM), the interaction between islet-resident macrophages (IRMs) and β cells is a key step in the initiation and maintenance of autoimmune responses. In 2020, Pavel et al. used single-cell transcriptomic analysis to demonstrate that IRMs undergo dynamic transcriptional reprogramming during T1DM progression. In the early stage, there is a gradual expansion of pro-inflammatory subsets (such as Mac-3 (CXCL9)), which highly express IFN-γ–responsive genes (e.g., CXCL9 and IL-2β) and MHC-II molecules. These features suggest that IRMs may activate T cells via antigen presentation. In contrast, the late stage is marked by a reduction in anti-inflammatory subsets (such as Mac-4 (Prdx1)), which have the ability to phagocytose apoptotic β-cell debris (50). Multiple studies have indicated that IRMs contribute to autoimmune destruction of β cells through antigen presentation, secretion of pro-inflammatory cytokines, and other mechanisms. Myeloid cells within the islets—including macrophages and dendritic cells—act as a bridge connecting stressed β cells and the adaptive immune system. Dysregulation of their function may trigger autoimmune activation (20).

#### 5.1.1 Antigen presentation and immune activation

As resident antigen-presenting cells within the islets of Langerhans, IRMs initiate and amplify autoimmune responses in T1DM by presenting self-antigens via MHC class II molecules and providing co-stimulatory signals such as CD80 and CD86 to activate autoreactive T cells. This drives Th1 polarization and recruits additional immune cells to infiltrate the islets. Experimental studies have shown that targeted depletion of IRMs in the islets of NOD mice completely blocks insulitis and the progression of diabetes (88), and adoptive transfer of T cells from these mice fails to induce disease in recipient animals (89), indicating that IRMs are essential for initiating T cell–mediated β-cell destruction. In mouse models, IRMs express high levels of MHC-II molecules such as I-A^g7^ (47, 66), allowing them to process and present self-antigens released by β-cells, including insulin and glutamic acid decarboxylase (GAD) (10, 90). Research by Emil R. Unanue and Xiaoxiao Wan in 2019 revealed that IRMs can process β-cell antigens under physiological conditions and activate CD4^+^ T cells via MHC-II, suggesting that this mechanism may breach immune tolerance and trigger autoimmunity in genetically susceptible individuals (3). Moreover, the antigen-presenting capacity of IRMs is closely linked to the T1DM-susceptible allele I-Ag7 in mice (66), whereas in humans, susceptibility is strongly associated with HLA class II loci, particularly HLA-DR and HLA-DQ (91), which are considered the most influential genetic risk factors for T1DM, as they determine the immune system’s ability to recognize islet self-antigens (92).

Recent human studies indicate that β-cells are not merely passive targets but may actively participate in antigen presentation by upregulating HLA class I molecules—especially HLA-ABC and HLA-F—during T1DM development. This phenomenon has been observed consistently in three independent cohorts (nPOD, DiViD, and newly diagnosed UK T1DM cases) and is regarded as a hallmark immunopathological feature of T1DM. HLA-I expression is persistently elevated in β-cell–rich islets and strongly correlates with the transcription factor STAT1, but not with the canonical HLA-I regulator NLRC5, implicating STAT1 as the key driver (93).

Beyond antigen presentation, IRMs interact with CD28 on T cells through their co-stimulatory molecules CD80 and CD86, enhancing TCR signal strength, promoting Th1 differentiation, and stimulating the release of pro-inflammatory cytokines such as IFN-γ and IL-2 (94). This cooperative signaling not only activates cytotoxic CD8^+^ T cells but also amplifies immune cell infiltration into the islets, creating a positive feedback loop that escalates autoimmune destruction. In humans, upregulated HLA-I expression may enhance recognition of β-cell antigens by CD8^+^ cytotoxic T cells, thereby contributing to early-stage autoimmunity in T1DM (93). Intravital imaging has shown that IRMs are spatially co-localized with T cell extravasation sites and secrete chemokines that mediate T cell retention and trans-endothelial migration; depletion of macrophages significantly reduces T cell infiltration into the islets (95). It is noteworthy, however, that while specific clearance of IRMs using clodronate liposomes markedly reduces CD8^+^ T cell migration into the islets, it does not completely block infiltration (96), suggesting that IRMs play a critical but non-exclusive role in initiating T cell trafficking. β-cells themselves may provide the initial activating signals, with IRMs serving to amplify and regulate the immune response.

A novel *in vitro* model known as “reformed islets” offers a high-fidelity platform for functional studies of IRMs. This model preserves the complete immune microenvironment, including Iba-1^+^ macrophages and CD8^+^/CD4^+^ T cells, and maintains expression of macrophage markers (CD45, Adgre1) and some M2-associated genes (e.g., Arg1) (21). Targeting IRM-mediated pathways—such as blocking antigen presentation or co-stimulatory signaling—may represent a promising therapeutic approach for halting T1DM progression, and the reformed islet model provides a critical tool for elucidating the underlying immune regulatory networks.

#### 5.1.2 Proinflammatory cytokine secretion and polarization

The phenotypic polarization of IRMs plays a decisive role in the progression of T1DM. In primary human T1DM tissues, clustered CD68^+^ macrophages have been observed within the islets, expressing high levels of IL-1β and TNF-α, indicative of M1-type activation. Concurrently, β-cells exhibit molecular signatures of endoplasmic reticulum (ER) stress and upregulation of heat shock proteins (HSPs), suggesting a spatial and pathological correlation between pro-inflammatory macrophages and β-cell dysfunction. Furthermore, in pancreatic sections from children within one year of diabetes onset, IL-1β expression was found to colocalize with regions of insulin loss, supporting the hypothesis that M1-polarized macrophages contribute to early-stage β-cell destruction (97). In 2015, Lindsey E. Padgett et al. confirmed that NADPH oxidase (NOX)–derived reactive oxygen species (ROS) promote IRM polarization toward a pro-inflammatory M1 phenotype, characterized by increased TNF-α and IL-1β secretion. In contrast, IRMs in NOX-deficient NOD mice exhibited an anti-inflammatory M2 phenotype, with high expression of arginase-1 (98). Recent studies further indicate that β-cells respond heterogeneously to Ca²^+^ overload, influencing macrophage polarization: some β-cells undergo apoptosis and release pro-inflammatory signals (e.g., casp-3, nfkbiaa), while others dedifferentiate (e.g., aldh1a3 expression), escaping death but losing functionality. This heterogeneity may differentially shape macrophage polarization via distinct signaling cues such as IL-1β and TNF-α (45). Activated macrophages positively correlate with the upregulation of β-cell dedifferentiation markers, including Ngn3 and Oct4 (50, 99, 100). However, the precise molecular mechanisms governing IRM–β-cell interactions remain incompletely defined and likely involve multiple dimensions of crosstalk, including direct cell–cell contact, soluble mediators, and exosome-mediated microRNA transfer.

In the human T1DM pathological context, pro-inflammatory cytokines such as IL-1β and IFN-γ, or viral infections, can induce ER stress in β-cells, leading to the release of DAMPs (damage-associated molecular patterns) that recruit macrophages into the islet microenvironment (101–103). Activated IRMs further secrete TNF-α and IL-1β, which activate the NF-κB pathway in β-cells, triggering apoptosis—an effect validated in reconstituted *in vitro* islet models (21, 104). IRMs exhibit notable functional heterogeneity: IL-1β can inhibit insulin synthesis and induce β-cell pyroptosis via the iNOS/NO pathway (48), while IFN-γ stimulates high expression of PD-L1 on β-cells, which interacts with PD-1 on Treg cells to establish a protective immune checkpoint—a phenomenon observed in both humans and mice (105, 106). This bidirectional balance between inflammation and immunoregulation suggests that targeting macrophage polarization—for instance, by promoting the M1-to-M2 transition—may represent a novel therapeutic strategy. Additionally, disrupting key pathways such as TLR4/NLRP3 or modulating the metabolic microenvironment, including the CXCL16–OxLDL axis, may help break the vicious inflammatory cycle and offer multidimensional entry points for treatment.

#### 5.1.3 Neuroimmune modulation and local inflammation

The sympathetic nervous system contributes to T1DM pathogenesis by modulating the function of IRMs. In a 2020 study, Gustaf Christoffersson et al. demonstrated that intra-islet sympathetic nerves activate IRMs through α1-adrenergic receptors, thereby promoting the local infiltration of CD8^+^ T cells and macrophages. Notably, either surgical denervation or pharmacological blockade of the receptor significantly delayed the onset of diabetes (5). This neuroimmune interaction provides a mechanistic explanation for the focal distribution of islet lesions observed in T1DM, highlighting the role of sympathetic nerve–mediated signaling in shaping the spatial pattern of autoimmune infiltration.

#### 5.1.4 Oxidative stress and immune microenvironment

In 2024, Neetu Srivastava et al. uncovered a novel mechanism by which IRMs maintain islet microenvironmental homeostasis through CXCL16-mediated clearance of oxidized low-density lipoprotein (OxLDL). In NOD mice, deletion of CXCL16 led to OxLDL accumulation, which impaired the survival and effector functions of transitional CD8^+^ T cells (Tex^int) within the islets, thereby delaying diabetes onset (10). In the same year, Jadie Y. Moon and colleagues further emphasized that IRMs support the persistence and differentiation of pathogenic CD8^+^ T cells via the CXCL16–OxLDL axis; however, this mechanism has yet to be validated in human samples (107).

Collectively, these findings reveal a shift in IRM function during T1DM—from homeostatic immune regulators to proinflammatory effectors—facilitating β cell destruction through multiple mechanisms. Strategies aimed at preserving the regulatory phenotype of IRMs or targeting key pathways (e.g., Siglec-E, CXCL16, CSF-1R) may offer promising avenues for preventing or treating T1DM.

### 5.2 Mechanisms of IRM–β cell interaction in the development of T2DM

**Figure 1:**
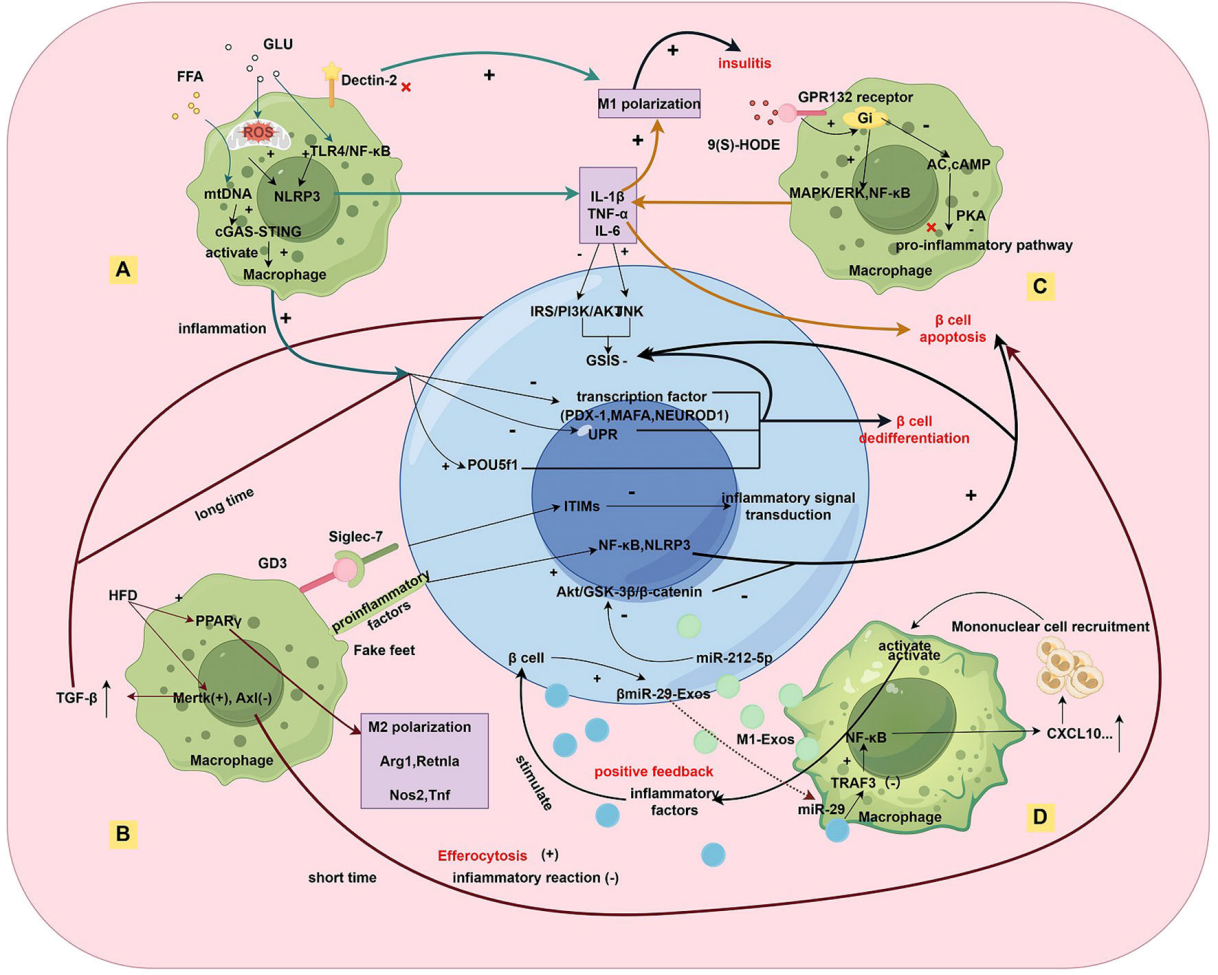
Multidimensional Regulatory Network of Macrophage-β-cell Interactions in the Inflammatory Islet Microenvironment of T2DM. Under metabolic stress **(A)**, hyperglycemic/hyperlipidemic environments activate the NLRP3 inflammasome in macrophages via TLR4/NF-κB, mtROS, and cGAS-STING pathways, leading to IL-1β/TNF-α secretion that impairs β-cell insulin secretion. The lipid peroxidation product 9(S)-HODE binds to the GPR132 receptor, activating the Gi/MAPK/NF-κB pathway and exacerbating M1 polarization. In macrophage phenotype dynamics **(B)**, a high-fat diet (HFD) induces M2 polarization (upregulated Arg1/Retnla) via PPARγ signaling and TAM receptor imbalance (Mertk↑/Axl↓), promoting efferocytosis while increasing TGF-β secretion, which chronically suppresses β-cell function. Macrophages exhibit a mixed pro-inflammatory (M1) and pro-fibrotic (M2-like) phenotype. Regarding direct contact and exosome-mediated interactions **(C)**, macrophages transfer IL-1β to β-cells via pseudopodia, activating NLRP3/NF-κB pathways (aggravated by Siglec-7 downregulation). In exosome crosstalk, M1-derived exosomes (M1-Exos) deliver miR-212-5p to inhibit β-cell Akt/GSK-3β signaling, while β-cell-derived miR-29 exosomes (βmiR-29-Exos) activate macrophage NF-κB/CXCL10, recruiting monocyte infiltration and forming a pro-inflammatory feedback loop. In epigenetic and transcriptional dysregulation **(D)**, Kdm5a-mediated H3K4 demethylation suppresses inflammatory genes to preserve β-cell function, while its deficiency impairs GSIS. Chronic inflammation downregulates transcription factors (PDX-1/MafA) and upregulates progenitor markers (Nanog/Pou5f1), inducing β-cell dedifferentiation and insulin secretion failure.

In contrast to T1DM, where adaptive immunity plays a central role, inflammation in obesity and T2DM is primarily driven by innate immune cells such as macrophages. As shown in **Figure 1**, these macrophages contribute to disease progression by promoting β-cell proliferation through signaling molecules like PDGF, while simultaneously impairing insulin secretion through various mechanisms (8). In T2DM and obesity-related inflammation, mechanisms by which hyperglycemia induces pro-inflammatory macrophage polarization have received considerable attention. The resulting inflammatory microenvironment not only exacerbates β-cell dysfunction but also perpetuates disease progression through dynamic shifts in macrophage phenotypes.

Multiple studies have demonstrated increased infiltration of macrophages in the islets of T2DM patients, with these cells contributing to β-cell dysfunction primarily via pro-inflammatory polarization (108). As early as 2002, Kathrin Maedler et al. observed that IL-1β, detected in pancreatic sections from five T2DM patients, may amplify local immune responses (109). In 2014, Helena Cucak and colleagues reported a significant increase in islet macrophage numbers in db/db diabetic mice (averaging 12 macrophages per islet), compared to only 2 per islet in control animals. These infiltrating macrophages predominantly displayed an M1-like pro-inflammatory phenotype, characterized by high expression of galectin-3 and low expression of CD80/CD86 (4). Furthermore, in mouse models, islet macrophages undergo a continuous shift from early pro-inflammatory phenotypes to later fibrotic profiles, rather than following a simple M1/M2 binary classification. Complementary findings by Ying et al. showed no significant change in the M1/M2 ratio of intra- and peri-islet macrophages in obese mice, suggesting that IRMs in T2DM may undergo mixed or hybrid phenotypic remodeling (7).

#### 5.2.1 Metabolic stress and chronic inflammation

Under metabolic stress, hyperglycemia induces the accumulation of mitochondrial reactive oxygen species (mtROS), which activates the TLR4/NF-κB signaling pathway and subsequently triggers the activation of the NLRP3 inflammasome. This signaling cascade has been well documented in murine models (110). Activated NLRP3 inflammasomes in islet macrophages promote the secretion of pro-inflammatory cytokines such as IL-1β, TNF-α, and IL-6—findings primarily based on experiments using mouse peritoneal macrophages and isolated islet cells (110, 111). Although systematic confirmation of this entire signaling pathway in human tissues is still lacking, preliminary evidence from human islets indicates that IL-1β can elicit a similar inflammatory response, suggesting a degree of conservation across species (112). These inflammatory cytokines act on β-cells by activating the JNK pathway and inhibiting the IRS/PI3K/Akt axis, thereby impairing glucose-stimulated insulin secretion (GSIS). This effect is especially pronounced in β-cell–specific IL-1 receptor antagonist (IL-1Ra) knockout mice (111). Consistent with this, *in vitro* studies using human primary islets and the ENDOC-βH1 β-cell line have shown that IL-1β reduces GSIS in human β-cells (110).

In addition, high-fat diets (HFDs) promote the accumulation of saturated fatty acids in islets, leading to mitochondrial dysfunction and the leakage of mitochondrial DNA (mtDNA) into the cytosol. This leakage activates the cGAS–STING signaling pathway in macrophages, further exacerbating inflammatory responses (113). In mouse models, macrophage accumulation and chronic inflammation suppress the expression of β-cell identity transcription factors—such as Pancreatic and Duodenal Homeobox 1 (PDX-1), MafA, and Neurogenic Differentiation 1 (NEUROD1)—downregulate adaptive unfolded protein response (UPR) genes, and upregulate progenitor markers like Nanog and POU5F1, thereby promoting β-cell dedifferentiation and loss of insulin secretory function. This establishes a vicious cycle of inflammation and metabolic dysregulation (114). In db/db mice, depletion of macrophages partially restores the expression of β-cell identity and UPR genes, underscoring the critical role of macrophages in mediating β-cell dysfunction (115). However, these mechanisms have not yet been definitively confirmed in primary human islet tissues. Although downregulation of PDX-1 and MafA and UPR gene disruption have been observed in T2DM patient islets, whether these changes are macrophage-mediated and involve the cGAS–STING pathway remains to be systematically validated (113–115).

#### 5.2.2 Metabolic and epigenetic regulation

Islet macrophages regulate β-cell function through dynamic metabolic reprogramming and shifts in polarization states, involving multilayered regulatory networks. Under homeostatic conditions, IRMs maintain a balance between aerobic glycolysis and oxidative phosphorylation, sustaining mitochondrial function by suppressing key glycolytic enzymes such as pyruvate dehydrogenase kinase (PDK). However, in high-fat diet (HFD)–induced models, IRMs switch to fatty acid oxidation (FAO), promoting M2-type polarization. In such models, Kdm5a-mediated histone H3K4 demethylation represses pro-inflammatory genes in IRMs while enhancing the expression of reparative factors, thereby preserving β-cell function. Inhibition of Kdm5a significantly impairs GSIS (116).

HFD also activates the PPARγ signaling pathway, driving IRMs toward an anti-inflammatory, pro-fibrotic M2 phenotype, as evidenced by upregulation of M2 markers (e.g., Arg1, Retnla) and suppression of M1 markers (e.g., Nos2, Tnf) (116–120). Concurrently, HFD disrupts the balance of TAM (Tyro3, Axl, Mertk) receptor signaling in IRMs, specifically upregulating Mertk (critical for efferocytosis) while downregulating Axl (involved in tissue repair). This shift enhances macrophage clearance of apoptotic cells (59, 116, 121–124), but also increases TGFβ secretion, which in turn impairs insulin secretory pathways in β-cells, such as PI3K/Akt and calcium signaling (116, 125, 126). While efferocytosis can be protective in the short term by mitigating inflammation, prolonged accumulation of inhibitory factors like TGFβ can induce β-cell dedifferentiation, characterized by downregulation of transcription factors such as PDX1 and MAFA, ultimately leading to insulin synthesis and secretory failure (44, 116, 127).

In HFD-induced T2DM models, the lipid peroxidation product 9(S)-HODE activates GPR132 receptors on islet macrophages, triggering Gi protein–coupled signaling that suppresses adenylate cyclase (AC) activity, reduces intracellular cAMP levels, and releases PKA-mediated inhibition of pro-inflammatory pathways. Simultaneously, the Giβγ subunit activates MAPK/ERK and NF-κB pathways, enhancing IL-1β and TNF-α production and promoting M1-type macrophage polarization. This inflammatory cascade contributes to islet inflammation and β-cell dysfunction. A GPR132 antagonist, NOX-6-18—rationally designed based on receptor structure—effectively blocks this axis and significantly improves metabolic outcomes and islet pathology in HFD-fed mice, offering a novel therapeutic strategy targeting lipid–GPCR signaling in diabetes (128). Furthermore, the C-type lectin receptor Dectin-2 has been identified as a key regulator of macrophage polarization; its deletion strongly promotes M1-type polarization and accelerates diabetes progression (129, 130).

#### 5.2.3 Direct cell–cell contact

During the pathological progression of T2DM, direct contact and material exchange between islet macrophages and β-cells are considered key mechanisms driving islet dysfunction. Using pancreatic tissues from T2DM donors, Gitanjali Dharmadhikari et al. discovered that macrophages and β-cells may interact through Siglec-7 receptors on β-cells and their ligand GD3 on macrophages, thereby modulating their respective activation states. Siglec-7 is an inhibitory receptor expressed on β-cells that suppresses inflammatory signaling through immunoreceptor tyrosine-based inhibitory motifs (ITIMs) in its cytoplasmic domain. In T2DM, Siglec-7 expression is markedly reduced, which increases β-cell sensitivity to inflammatory stimuli. Overexpression of Siglec-7 was shown to inhibit NF-κB activation, decrease pro-inflammatory cytokine secretion, and protect β-cell viability and function by restoring insulin secretion (131). Moreover, immunohistochemistry and confocal microscopy studies by Ehses et al. revealed a significant increase in the number of macrophages in close proximity to β-cells in T2DM islets. These macrophages often extend pseudopodia that make direct contact with β-cell surfaces, enabling more efficient delivery of inflammatory cytokines (such as IL-1β) and other signaling molecules. This activates pro-inflammatory pathways within β-cells, including NF-κB and the NLRP3 inflammasome, leading to functional decline and apoptosis (61).

These contact-dependent mechanisms collectively contribute to the formation of a pro-inflammatory islet microenvironment, perpetuating a vicious cycle in T2DM progression and presenting potential molecular targets for anti-inflammatory interventions in islet inflammation.

#### 5.2.4 IRM- and β cell–derived exosomes

Studies have shown that in high-fat diet (HFD)–fed mice, exosomes released by M1-polarized islet-resident macrophages (M1-Exos) can be internalized by β-cells, subsequently inhibiting insulin secretion. This suppressive effect is mediated by the microRNA miR-212-5p, which is significantly upregulated in M1-Exos. These exosomes transfer miR-212-5p to β-cells, where it targets the SIRT2 gene, thereby inhibiting the Akt/GSK-3β/β-catenin signaling pathway and impairing GSIS (69). Targeting macrophage-derived exosomes or their miRNA cargo (such as miR-212-5p) may represent a promising therapeutic strategy for T2DM. In mice, β-cells can also release exosomes containing miR-29 (βmiR-29-Exos) under metabolic stress, such as a high-fat diet. Once internalized by circulating monocytes and IRMs, these exosomes suppress TRAF3 expression and activate the NF-κB pathway, thereby inducing chemokines such as CXCL10 that recruit additional monocytes into the islet. The activated macrophages further secrete IL-1β and other inflammatory cytokines, which in turn stimulate β-cells to release more miR-29–laden exosomes, forming a positive feedback loop (132). This reciprocal interaction expands local islet inflammation into systemic inflammation, driving peripheral insulin resistance and β-cell dysfunction, and ultimately accelerating the progression of T2DM.

## 6 Analysis of consistencies and differences

### 6.1 Shared features across different diabetes models

Both T1DM and T2DM models display notable pathological similarities in the interactions between macrophages and β-cells, as summarized in **Table 1**. First, increased infiltration of macrophages within pancreatic islets is a common observation in both disease models (4, 64).

**Table 1:** Shared features of T1DM and T2DM models.

Shared Feature	Mechanism / Function	Key Findings	References
Increased Macrophage Infiltration	Remodeling of the islet immune microenvironment during diabetes progression	Significant increase in islet macrophage numbers in both T1DM (NOD mice) and T2DM (obesity models)	(4, 64)
Pro-inflammatory (M1-like) Polarization	Secretion of TNF-α, IL-1β, and other pro-inflammatory cytokines	Dominance of M1 phenotype in NOD mice; M1 macrophage accumulation observed in T2DM	(8, 98)
Close Cell–Cell Interaction	Sensing β cell secretory status and viability via direct contact	Spatial proximity between macrophages and β cells influences disease progression	(21, 69)
Sensitivity to Oxidative Stress	NADPH oxidase–derived ROS regulate macrophage polarization	Oxidative microenvironments modulate autoimmunity; β cell stress activates innate immunity	(10, 20, 98)

In both types of diabetes, islet macrophages predominantly exhibit a pro-inflammatory phenotype. In NOD mice, infiltrating macrophages display an M1-like profile and secrete large quantities of pro-inflammatory cytokines such as TNF-α and IL-1β (98). Similarly, in T2DM mouse models (e.g., db/db mice), Helena Cucak et al. reported that islet macrophages primarily adopt an M1-like phenotype (4).

The mechanisms through which macrophages interact with β-cells are also consistent across both models. In a 2021 review, Cristina Cosentino and Romano Regazzi emphasized that macrophages can closely monitor β-cell secretory capacity and viability via direct cell–cell contact—an anatomical arrangement shared by both T1DM and T2DM models (2). Bin Qian et al. further demonstrated that M1-polarized macrophages influence β-cell function through both cytokine-dependent and -independent mechanisms, which are common to both types of diabetes (69).

Importantly, macrophages in both models exhibit heightened sensitivity to oxidative stress. In 2024, Neetu Srivastava et al. showed that increased oxidative stress within the islet microenvironment can alter macrophage function and modulate autoimmune responses (10). This aligns with findings by Lindsey E. Padgett et al., who demonstrated that NOX-derived reactive oxygen species (ROS) promote M1 polarization in macrophages (98). These results collectively suggest that oxidative stress is a key mechanistic link in macrophage–β-cell crosstalk in both T1DM and T2DM. Supporting this notion, Henner Zirpel and Bart O. Roep proposed that β-cell dysfunction and cellular stress may serve as initiating events that activate the innate immune system in diabetes (20).

### 6.2 Major differences between models

Despite common features, macrophage–β-cell interactions display model-specific characteristics across different forms of diabetes, particularly in terms of macrophage subset distribution, functional phenotypes, and modes of impact on β-cells, as summarized in **Table 2**.

**Table 2:** Major differences between T1DM and T2DM models.

Dimension of Difference	T1DM Models (e.g., NOD Mice)	T2DM Models (e.g., Obesity-Associated Models)	References
**Macrophage Origin and Renewal**	Primarily embryonic-derived; depletion significantly suppresses autoimmunity (via CSF-1R blockade)	Bone marrow-derived monocytes replace embryonic origin; local proliferation driven by PDGF signaling	(58, 133)
**Core Functional Mechanisms**	Initiation of autoimmunity: - Antigen presentation (CSF-1R–dependent) - CXCL16/OxLDL axis maintains oxidative stress and supports CD8^+^ T cell survival	Metabolic regulation and inflammation: - Pro-inflammatory cytokines impair β cell function - Involved in vascular remodeling during compensatory hyperinsulinemia	(10, 40, 133)
**Signaling Pathway Features**	Sympathetic α1-adrenergic receptor activates macrophages (Christoffersson et al.); CXCL16 enhances CD8^+^ T cell effector functions	PDGF signaling drives β cell proliferation; contact-dependent suppression of β cell function	(5, 8)
**Effects on β Cells**	Triggers autoimmune responses: - CD4^+^/CD8^+^ T cell infiltration - Activation of autoreactive T cells	Suppresses insulin secretion; promotes compensatory β cell proliferation	(40, 69)
**Therapeutic Targets**	CSF-1R, CXCL16/OxLDL axis, sympathetic α1-adrenergic receptor	PDGF pathway, oxidative stress signaling, macrophage–β cell contact mechanisms	(10, 20, 58, 69, 98, 133)

In obesity-associated T2DM models, Wei Ying et al. reported that islet macrophages expand primarily via local proliferation and promote β-cell proliferation through PDGF signaling, while concurrently impairing insulin secretion (8). Ziyuan Ma and Christiane Ruedl further showed that although the renewal kinetics of islet-resident macrophages remain unchanged under obese diabetic conditions, embryonically derived macrophages are gradually replaced by bone marrow–derived monocytes (58).

By contrast, in NOD mice (a model of T1DM), macrophages primarily function to initiate autoimmune responses. Javier A. Carrero et al. demonstrated that CSF-1R antibody–mediated depletion of islet-resident macrophages in NOD mice significantly reduced early infiltration of CD4^+^ T cells and dendritic cells, impaired presentation of insulin epitopes, and ultimately lowered the incidence of diabetes (133). Xiaoxiao Wan et al. found that IRMs in NOD islets exhibit high CXCL16 expression, which facilitates the clearance of OxLDL and maintains a redox environment that supports the survival and effector function of CD8^+^ T cells (Tex^int) (10). Gustaf Christoffersson and colleagues revealed a unique neuroimmune mechanism in which sympathetic nerve signals act through α1-adrenergic receptors on islet macrophages, promoting the infiltration of CD8^+^ T cells and macrophages into the islets (5).

Interestingly, Manesh Chittezhath et al. observed across multiple models that islet macrophages play a role in vascular remodeling during the phase of compensatory hyperinsulinemia, and that dysfunction in this process may accelerate diabetes progression (40). Collectively, these studies underscore the distinct roles of macrophages across disease contexts: T1DM models emphasize their role in initiating autoimmunity, while T2DM models highlight their pro-inflammatory and metabolic regulatory functions.

## 7 Limitations and critical analysis

### 7.1 Current research shortcomings

Despite significant advances in understanding IRM–β-cell interactions, several technical limitations and knowledge gaps remain. In terms of characterizing macrophage heterogeneity, although Ziyuan Ma and Christiane Ruedl (2022) successfully distinguished three pancreatic exocrine macrophage subsets using Tim-4 and MHC II markers (58), the phenotypic classification of intra-islet macrophages remains underdeveloped. While islet-resident macrophages are generally considered homogeneous, Wei Ying et al. (2019) identified two functionally distinct subsets within the islets of obese mice (7), suggesting that existing marker systems may be insufficient to capture the full spectrum of macrophage diversity.

There are also limitations in tracking the dynamic interactions between IRMs and β-cells. Bernd H. Zinselmeyer et al. (2018) utilized two-photon microscopy to observe macrophage pseudopodia directly contacting blood vessel lumens (66), but this technique is constrained in its ability to follow single-cell behaviors over extended time periods. Cristina Cosentino and Romano Regazzi (2021) emphasized that macrophages dynamically respond to β-cell secretory activity (2); however, current technologies lack the spatiotemporal resolution needed to delineate the molecular dynamics of these interactions *in vivo*.

Methodological limitations also exist in the study of phagocytosis. Kristel Parv et al. (2021) compared phagocytic activity between endocrine and exocrine macrophages using ex vivo assays (59), but *in vitro* culture may alter the native state of these cells. Henner Zirpel and Bart O. Roep (2021) highlighted that studies on human islet macrophages still rely heavily on postmortem tissues (20), which hinders real-time functional assessment during disease progression. Moreover, existing models do not effectively distinguish the functional roles of embryonically derived versus bone marrow–derived macrophages (7, 58), despite evidence that these two populations may exert fundamentally different effects on β-cell homeostasis.

### 7.2 Challenges in clinical translation

Although animal studies have significantly contributed to elucidating IRM–β cell mechanisms, translating these findings to clinical settings still faces several challenges. These are discussed from three aspects below.

#### 7.2.1 Species differences and human disease heterogeneity

As mentioned in the introduction, regulatory mechanisms observed in animal models may not fully capture the complexity of human disease. In 2023, Eunwon Lee et al. found that extracellular vesicles (EVs) derived from 3D-cultured human umbilical cord blood mesenchymal stem cells (3D hUCB-MSC-EVs) promote M2-type macrophage polarization and protect β-cells (134), yet the intricate microenvironment of the human islet may lead to variable efficacy. Henner Zirpel et al. (2021) emphasized the scarcity of functional studies on antigen-presenting cells within human islets (20), which limits the translational relevance of findings from rodent models. Potential solutions include the development of humanized mouse models and organoid-based culture systems that better recapitulate the human islet milieu.

#### 7.2.2 Targeted delivery and safety concerns

Precise intervention targeting specific macrophage subsets remains technically challenging. In 2023, Jia-Le Wang’s team employed structural biology approaches to design a GPR132 antagonist for macrophage reprogramming (128), yet achieving high local drug concentrations within islets remains an unresolved issue. In 2024, Neetu Srivastava et al. demonstrated that CXCL16 deficiency alters the oxidative landscape of islets (10), raising concerns that systemic interventions may disrupt metabolic homeostasis. Targeted delivery strategies—such as nanoparticle-based systems or gene expression driven by macrophage-specific promoters—may enhance safety and therapeutic efficacy.

#### 7.2.3 Balancing immune modulation and infection defense

Maintaining a balance between immunosuppression and infection defense is a central challenge in clinical translation. In 2016, Thomas B. Thornley et al. showed that IRMs can induce regulatory T cells (Tregs) (6), but excessive suppression of immune functions may increase infection risk. Henner Zirpel (2021) emphasized the dual roles of human islet macrophages in both maintaining homeostasis and promoting pathology, underscoring the need for fine-tuned regulation (20). Promising strategies may include reversible immunomodulatory approaches—such as optogenetically controlled drug release systems—and the development of personalized immune-monitoring platforms to ensure precise treatment responses.

## 8 Research trends and future directions

### 8.1 Novel therapeutic strategies

In recent years, research on IRM–β cell interactions in diabetes has uncovered multiple potential therapeutic targets. Among these, macrophage polarization regulation and modulation of intercellular communication networks represent two of the most promising directions.

#### 8.1.1 Breakthrough in GPR132-targeted therapy

In 2023, Jia-Le Wang et al. conducted a comprehensive analysis of lipid–transmembrane receptor signaling in IRMs and identified the endogenous 9(S)-HODE–GPR132–Gi signaling axis as a central regulator of macrophage reprogramming. Using cryo-electron microscopy, they resolved the structural interaction between GPR132 and its endogenous agonist, which enabled the rational design of a highly selective GPR132 antagonist. *In vivo* experiments demonstrated that GPR132 deficiency significantly ameliorates metabolic disturbances induced by high-fat diet feeding, establishing a strong rationale for developing selective GPR132 modulators as potential therapies for type 2 diabetes (128). Notably, GPR132 antagonism not only alters macrophage polarization but also improves the inflammatory status of the islet microenvironment, positioning it as a dual-action target.

#### 8.1.2 Strategies for regulating macrophage polarization

Multiple studies have demonstrated that promoting macrophage polarization toward the M2 phenotype via exogenous interventions can effectively preserve β cell function. Eunwon Lee et al. found that extracellular vesicles (EVs) secreted by three-dimensional cultured human umbilical cord blood mesenchymal stem cells (3D hUCB-MSCs) are enriched with microRNAs that promote M2 polarization. These EVs increased the proportion of M2 macrophages in islets, suppressed proinflammatory cytokine expression, and enhanced glucose-stimulated insulin secretion. Furthermore, they preserved β cell identity by upregulating key transcription factors such as Pdx1 and FoxO1 (134). Similarly, Paul J. Belmonte et al. demonstrated that overexpression of ST8Sia6 enhances production of Siglec-E ligands, significantly reducing hyperglycemia in STZ-induced diabetic models, offering new immunoregulatory strategies to modulate macrophage function (135).

#### 8.1.3 Targeting intercellular communication networks

The complex network of cellular communication within the islet microenvironment also represents a critical intervention point. Thomas B. Thornley et al. found that IRMs in non-autoimmune-prone mice exhibit a regulatory phenotype that potently induces FoxP3^+^ Tregs (6). However, this function is disrupted by TLR4 activation or during diabetes progression. These findings suggest that preserving the immunoregulatory phenotype and abundance of IRMs may offer a new approach to T1DM prevention. Furthermore, Wei Ying’s group demonstrated that in obesity and T2DM, islet macrophages promote β-cell proliferation via PDGF signaling but simultaneously impair insulin secretion (8). Targeting such specific signaling pathways could help resolve both quantitative and functional defects in β-cells.

Based on current evidence, future therapeutic strategies should prioritize the following directions: (1) rational design of small molecules targeting novel receptors such as GPR132, with attention to tissue selectivity and long-term safety; (2) optimization of biotherapeutic production protocols, including standardization of 3D culture systems for EV generation (134); (3) development of combination therapies that simultaneously target macrophage polarization and β-cell protection (8, 135); and (4) engineering of spatiotemporally specific drug delivery systems to fine-tune the islet microenvironment (6). The success of these strategies will rely on deeper mechanistic understanding of IRM–β-cell interactions and rigorous preclinical validation.

### 8.2 Technological innovation directions

The application of cutting-edge technologies such as single-cell sequencing and intravital imaging is revolutionizing the study of IRM–β cell interactions. These tools allow for high-resolution dissection of dynamic cellular behaviors and molecular signaling at single-cell and spatial levels.

Single-cell RNA sequencing (scRNA-seq) has been instrumental in identifying the heterogeneity of pancreatic macrophages. Ziyuan Ma and Christiane Ruedl (2022) found that IRMs exhibit distinct renewal kinetics during obesity and aging (58). Further scRNA-seq studies can uncover transcriptional signatures of different macrophage subtypes and their regulatory networks with β cells, such as specific cytokine–receptor pairs that influence β cell fate. Intravital imaging, especially two-photon microscopy, enables real-time observation of cellular interactions. In 2018, Bernd H. Zinselmeyer et al. used CX3CR-GFP transgenic mice to visualize macrophage–β cell–vascular contacts and the dynamic behavior of filopodia (66). This technique also allows monitoring of macrophage responses to glucose stimulation—such as filopodial retraction—as a direct readout of metabolic–immune crosstalk. Calcium imaging combined with pancreatic slice preparations has been used to study macrophage responsiveness to β cell signals. Jonathan R. Weitz et al. developed a mouse model expressing calcium sensors specifically in myeloid cells and showed that IRMs can sense ATP released from β cells in a glucose-dependent manner via purinergic receptors (64). This approach can be expanded to assess responses to other metabolites or inflammatory mediators. Flow cytometry and functional assays have been employed to assess phagocytic activity. Kristel Parv et al. (2021) showed that endocrine-resident macrophages exhibit stronger phagocytic function than their exocrine counterparts (59). Combining these with transcriptomic analysis can elucidate how local microenvironmental signals shape macrophage function. Henner Zirpel and Bart O. Roep (2021) proposed that single-cell technologies can help resolve the phenotypic and functional heterogeneity of human IRMs, which is still underexplored (20).

Future studies may integrate spatial transcriptomics and proteomics to retain *in situ* context while profiling molecular interactions. Combining live imaging with transcriptomic readouts will allow tracking of gene expression dynamics during cell–cell interactions, thereby offering new therapeutic targets for diabetes intervention.

## 9 Conclusion

The dynamic interplay between islet-resident macrophages (IRMs) and β cells plays a central role in the pathogenesis of diabetes. Accumulating evidence indicates that during disease progression, macrophage populations within the islet microenvironment undergo profound phenotypic shifts—from immunoregulatory states under physiological conditions to pro-inflammatory profiles tightly linked to β cell dysfunction.

In type 1 diabetes (T1DM), IRMs serve as key hubs of neuroimmune signaling. Activation of the sympathetic nervous system promotes immune cell infiltration into the islets via specific receptor pathways. Notably, macrophages that initially possess immune-suppressive properties lose their ability to induce Tregs as the disease progresses. In type 2 diabetes (T2DM), IRMs impair β cell function through multiple mechanisms, including cytokine secretion and regulation of lipid metabolism. As innate immune cells present since embryogenesis, IRMs maintain a stable spatial and functional relationship with β cells, enabling them to continuously monitor and influence insulin secretion and cell viability in real time.

Recent studies have identified several molecular mechanisms that mediate these cell–cell interactions, including lipid metabolism–related signaling pathways, chemokine-induced oxidative stress responses, and contact-dependent regulatory networks. Importantly, in obesity-associated islet inflammation, macrophages not only suppress β cell function via direct contact but also drive compensatory β cell proliferation through aberrant growth factor signaling.

These findings offer promising new directions for diabetes therapy. Strategies such as modulating macrophage polarization, blocking pathological signaling pathways, or reconstructing intercellular communication networks may effectively protect β cell function and restore metabolic homeostasis. Breakthroughs in this field not only deepen our understanding of the heterogeneous mechanisms underlying diabetes but also lay the theoretical foundation for developing precision therapies based on immunometabolic modulation.

## Glossary

IRMs: Islet-resident macrophages
T1DM: Type 1 diabetes mellitus
T2DM: Type 2 diabetes mellitus
EVs: Extracellular vesicles
β cells: Beta cells
HFD: High-fat diet
GPR132: G protein-coupled receptor 132
CXCL16: C-X-C motif chemokine ligand 16
OxLDL: Oxidized low-density lipoprotein
ROS: Reactive oxygen species
GSIS: Glucose-stimulated insulin secretion
Tregs: Regulatory T cells
IL-1β: Interleukin-1 beta
TNF-α: Tumor necrosis factor alpha
NF-κB: Nuclear factor kappa B
MAPK: Mitogen-activated protein kinase
JNK: c-Jun N-terminal kinase
PKA: Protein kinase A
cAMP: Cyclic adenosine monophosphate
PPARγ: Peroxisome proliferator-activated receptor gamma
SIRT2: Sirtuin 2
TRAF3: TNF receptor associated factor 3
STZ: Streptozotocin
PDGF: Platelet-derived growth factor
VEGF-A: Vascular endothelial growth factor A
IGF-1: Insulin-like growth factor 1
TGF-β: Transforming growth factor beta
ITIM: Immunoreceptor tyrosine-based inhibitory motif
MHC-II: Major histocompatibility complex class II
miRNA: MicroRNA
ATP: Adenosine triphosphate
SCID: Severe combined immunodeficiency
mtROS: Mitochondrial reactive oxygen species
mtDNA: Mitochondrial DNA
cGAS-STING: Cyclic GMP-AMP synthase–stimulator of interferon genes
NLRP3: NOD-like receptor pyrin domain-containing 3
TAM receptors: Tyro3, Axl, and Mertk receptors
Kdm5a: Lysine demethylase 5A
UPR: Unfolded protein response
PDX1: Pancreatic and duodenal homeobox 1
MAfA: v-Maf avian musculoaponeurotic fibrosarcoma oncogene homolog A
NEUROD1: Neurogenic differentiation 1
POU5f1: POU class 5 homeobox 1
CX3CR1: CX3C chemokine receptor 1
CCR2: C-C chemokine receptor type 2
CCL2: Chemokine (C-C motif) ligand 2
